# Efficacy of cinnamon bark oil and cinnamaldehyde on anti-multidrug resistant *Pseudomonas aeruginosa* and the synergistic effects in combination with other antimicrobial agents

**DOI:** 10.1186/s12906-016-1134-9

**Published:** 2016-06-01

**Authors:** Itsaraporn Utchariyakiat, Suvimol Surassmo, Montree Jaturanpinyo, Piyatip Khuntayaporn, Mullika Traidej Chomnawang

**Affiliations:** Department of Microbiology, Faculty of Pharmacy, Mahidol University, 447 Sri Ayudthaya Road, Rachathevi, Bangkok 10400 Thailand; National Nanotechnology Center (NANOTEC), National Science and Technology Development Agency, Khlong Luang, Thailand; Department of Manufacturing Pharmacy, Faculty of Pharmacy, Mahidol University, Bangkok, Thailand

**Keywords:** Cinnamaldehyde, Cinnamon bark oil, Multidrug-resistant *P. aeruginosa*, Synergy

## Abstract

**Background:**

The emergence of drug resistant pathogens becomes a crucial problem for infectious diseases worldwide. Among these bacteria, *Pseudomonas aeruginosa* is one of which highly resists to many currently used drugs and becomes a major concern in public health. Up till now, the search for potential antimicrobial agents has been still a challenge for researchers.

**Methods:**

Broth microdilution assay was used to determine minimum inhibitory concentrations (MICs) and minimum bactericidal concentrations (MBCs) of the essential oils and antibiotics against *P. aeruginosa.* Inhibition activity of the essential oils under vapor condition was examined to obtain the minimum inhibitory dose (MID). Time-kill assay included in this study was performed according to CLSI guideline. Bioautographic assay was used to detect active components of the essential oil. Synergistic effect with currently used antibiotics was further examined by checkerboard assay.

**Results and Discussion:**

In this study, a variety of essential oils were examined for anti-multidrug resistant *P. aeruginosa* (MDR-PA) activity, of which cinnamon bark oil showed the strongest antimicrobial activity against all clinical-isolated MDR-PA strains with MIC of 0.0562–0.225 % v/v and MBC of 0.1125–1.8 % v/v. Bioautographic results demonstrated that the active compounds of cinnamon bark oil were cinnamaldehyde and eugenol which showed strong inhibitory effect against *P. aeruginosa*. Interestingly, cinnamaldehyde, a major constituent of cinnamon bark oil, possessed stronger antimicrobial effect to *P. aeruginosa* than eugenol. Under gaseous condition, cinnamon bark oil and cinnamaldehyde showed antibacterial activity against MDR-PA strains with MID of 0.5–1 mg/L. Moreover, combination of cinnamon bark oil or cinnamaldehyde with currently used antibiotics was further studied by checkerboard assay to examine synergistic interactions on clinically isolated MDR-PA strains. Cinnamon bark oil and cinnamaldehyde combined with colistin demonstrated synergistic rates at 16.7 and 10 %, respectively.

**Conclusion:**

These results indicated that cinnamon bark oil and cinnamaldehyde might be active natural compounds which could be further examined as alternative treatment for multidrug-resistant *P. aeruginosa* infection.

## Background

*Pseudomonas aeruginosa* is a Gram-negative non-fermenting bacterium which has broad ecological adaptability and distribution. It is an opportunistic human pathogen, causing severe infections, especially in immunocompromised patients. This organism is classified as one of ‘hard to treat’ organisms and is one of the most antibiotic resistant bacteria in this century because of its adaptability to various conditions and multifactorial virulences; for example, endotoxin, exotoxin and proteolytic enzymes. In addition, *P. aeruginosa* has high intrinsic resistance to antibiotic and is able to develop new antibiotic resistance during treatment [1]. The ability of antibiotic resistance of *P. aeruginosa* is significantly due to low permeability of the cell wall, which restricts the uptake of antibiotics, and the genetic capacity to express a wide repertoire of resistance mechanisms, like efflux pumps and enzymes, which modify or degrade antibiotics and drug targets [2]. It was reported that the antibiotic tolerance of *P. aeruginosa* clinical isolates could enhance up to 6000-fold to ciprofloxacin and tobramycin [3–5].

Plant essential oils are potential sources of antimicrobial compounds and have been used in traditional medicine for many years [6–9]. Essential oils are natural compounds containing a complex mixture of odorous and volatile constituents. Essential oils have been observed to possess antibacterial, antifungal and anti-plasmodial activity. Several in vitro studies indicated that essential oils exhibited antibacterial agents against wide spectrum of pathogenic bacterial strains [10, 11]. For example, the leaves and flowers of *Achillea clavennae* in Croatia were investigated by disc diffusion method against Gram-positive and Gram-negative bacteria and showed strong activity against *Haemophilus influenzae* and *P. aeruginosa*. Also in India, *Aristolochia indica* containing β-carophyllene and α-humulene as major components was evaluated its antibacterial activity against six strains of bacteria by agar dilution method and demonstrated moderate activity [12]. Some essential oils such as oregano oil showed effects on multidrug-resistant *Escherichia coli* [13]. Antibacterial activities of the essential oils from *Ocimum gratissimum*, *Salvia officinalis* L, *Cymbopogon citratus* (DC) stapf were evaluated on the microorganisms isolated from urinary tract infection and all of these microorganisms except *P. aeruginosa* showed sensitivity to these essential oils [14]. Therefore, this study was aimed to determine the effect of essential oils and its major compounds against multidrug-resistant *P. aeruginosa*. In addition, the combination of essential oils and its major compounds with currently used antibiotics were also investigated.

## Methods

### Bacterial strains, essential oils and antimicrobial agents

*Pseudomonas aeruginosa* PAO1 and clinically isolated multidrug-resistant *P. aeruginosa* (MDR-PA) strains were cultured in tryptic soy broth (TSB) at 37 °C for 18 h. The overnight culture was adjusted to an optical density of 0.5 McFarland and was diluted to yield approximately 1.0 × 10^6^ cfu/ml.

Essential oils used in this study were betel vine oil (*Piper betle* Linn.*)*, cinnamon bark oil (*Cinnamomum zeylanicum*), citronella oil (*Cymbopogon nardus*), clove oil, clove leaf oil (*Syzygium aromaticum* Linn. (Merr. & Perry)), galangal oil (*Alpinia galangal* Linn.*)*, guava leaf oil (*Psidium guajava* Linn.), hairy basil oil (*Ocimum americanum* Linn.), holy basil oil (*Ocimum sanctum* Linn.), kaffir lime oil, kaffir lime leaf oil (*Citrus hystrix* DC*.*), lemon oil (*Citrus limonum* Linn.), lemongrass oil (*Cymbopogon flexuosus*), finger root oil (*Boesenbergia pandurata* (Roxb.) Schltr), plai oil (*Zingiber cassumunar* Linn.), sweet basil oil (*Ocimum bacilicum* Linn.), and turmeric oil (*Curcuma longa* Linn.) which were purchased from Thai-China Flavours and Fragrances Industry Co., Ltd, Thailand. Cinnamaldehyde was obtained from S.M. chemical supplies Co., Ltd, Thailand. The essential oils and cinnamaldehyde were kept in light protection container at 4 °C to prevent oxidative degradation. Tween 80 and 95 % ethanol were used as surfactant and co-surfactant to prepare essential oil solution. Essential oils and cinnamaldehyde were dissolved to appropriate concentration and stored at 4 °C before testing. Colistin sulfate was obtained from Acme Medical Co., Ltd, Thailand. Ceftazidime was purchased from Siam Bheasach Co., Ltd, Thailand. Imipenem and meropenem were purchased from L.B.S. Laboratory, Thailand. Piperacillin, and ciprofloxacin were purchased from Shionogi & Co, Japan.

### Antimicrobial susceptibility test

Determination of minimum inhibitory concentrations (MICs) and minimal bactericidal concentrations (MBCs) of the essential oils and antibiotics against *P. aeruginosa* PAO1 and clinical-isolated MDR-PA strains were performed by broth microdilution method as previously described [15]. MICs were obtained from the lowest concentration of active compound that inhibited bacterial growth. MBCs were further determined on Mueller-Hinton agar (MHA) and observed bacterial growth after 18–24 h incubation.

Inhibition activities under vapor condition of cinnamon bark oil, lemongrass oil, clove oil and cinnamaldehyde were examined on *P. aeruginosa* PAO1 and 19 MDR-PA isolates. The minimum inhibitory dose (MID) was defined as the lowest dose per unit space against bacterial growth. MID was expressed in term of weight per unit volume (mg/L air). Gaseous phase of essential oils were investigated on *P. aeruginosa* isolates according to Rodrigues FF et al. [16]. Briefly, essential oils were dissolved in dimethyl sulfoxide (DMSO) with ratio of 1:1 w/w, and dropped on paper disc. Agar plates were spread with bacterial suspension containing approximately 1.5 × 10^5^ cfu/ml before placing paper disc on the lid of the petri dish and incubated at 37 °C for 24 h under closed system.

### Time killing assay

Cinnamon bark oil and cinnamaldehyde were evaluated in terms of time killing effect on *P. aeruginos*a PAO1 and representative MDR-PA strain. Time-kill assay was performed according to CLSI guideline [17]. The concentrations of fosfomycin and carbapenems used in this assay to determine individual antimicrobial activity were selected from the Clinical Microbiology Procedures Handbook protocol [18]. Bacteria were inoculated in broth medium containing various concentrations of active compound as followed, 1xMIC, 2xMIC and 4xMIC. Treated bacterial cells were evaluated at a certain period of time by determining the cell viability and the bacterial growth compared to control.

### Thin Layer Chromatography and bioautography

Active components of essential oil were identified by thin-layer chromatography (TLC). Silica gel_GF254_ plates (Merck KGaA, Darmstadt, Germany) were developed in system of toluene: ethyl acetate at a ratio of 93:7 v/v according to Shahverdi et al. [19]. TLC paper coated with siliga gel was used as a stationary phase. They were developed in solvent system acting as mobile phase. The TLC plate was sprayed with 1 % v/v anisaldehyde sulfuric acid and heated at 110 °C for 5 min before visualized under visible and UV lights (254 and 366 nm). The constituents of compound were separated and calculated R_*f*_ values. TLC plates were performed in duplicate; one set was used as the reference chromatogram and another was used for bioautography. Bioautographic assay was performed to detect active components in essential oil [20]. Developed TLC plates were carefully dried to remove solvents, overlaid by agar seed, and incubated overnight at 37 °C. Inhibition zones were compared with R_*f*_ of the spots on the reference TLC plate.

### Synergistic activity

The interaction between essential oils and the antibiotics, which were ceftazidime (CAZ), piperacilin (PIP), colistin sulfate (COL), meropenem (MEM), doripenem (DOR), imipenem (IMI) and ciprofloxacin (CIP), were determined by combination assay against MDR-PA isolates. Bacteria were cultured in broth medium for 18 h before adjusted to 0.5 McFarland and the medium was diluted to obtain the concentration of 1.5 × 10^6^ cfu/ml. Combination assay were examined in 96-well plate, the first agent was diluted by two-fold dilution, followed by adding the second agent at various concentrations. Then, bacterial suspension was added for into each well and incubated at 37 °C for 24 h. The fractional inhibitory concentration index (FICI) was calculated for each couple of drug combination. FICI was a summation between MIC of drug A in combination divided by MIC of drug A alone (C_A_) and MIC of drug B in combination divided by MIC of drug B alone (C_B_).

$$ \begin{array}{l}{\displaystyle \sum \mathrm{FICI} = {\mathrm{FIC}}_{\mathrm{A}} + {\mathrm{FIC}}_{\mathrm{B}}}\\ {}\kern6.5em  = \left({\mathrm{C}}_{\mathrm{A}}/{\mathrm{MIC}}_{\mathrm{A}}\right) + \left({\mathrm{C}}_{\mathrm{B}}/{\mathrm{MIC}}_{\mathrm{B}}\right)\end{array} $$

Interpretation criteria of FICI were followed, if FICI ≤ 0.5 indicate synergy, FICI between >0.5 and ≤ 4 indicate indifferent, and FICI > 4 mean antagonism [21].

## Results and Discussion

### Antimicrobial activity of essential oils against multidrug resistant *P. aeruginosa*

Antibacterial activity was performed on 17 essential oils against *P. aeruginosa* PAO1.

Three plant oils which were clove oil, lemongrass oil, and cinnamon bark oil showed effective antimicrobial activity. Among those, cinnamon bark oil gave the strongest inhibition activity against *P. aeruginosa* wild-type strain with MIC 0.2 % v/v followed by clove oil and lemongrass oil with MICs of 1.8 % v/v. Other 14 essential oils exhibited low antimicrobial efficacy against *P. aeruginosa* with MIC and MBC ranges of more than 1.8–3.6 % v/v. Moreover, cinnamon bark oil was only essential oil which demonstrated the strongest activity with MBC at the lowest used concentration (0.2 % v/v). In addition, cinnamon bark oil, lemongrass oil and clove oil were further examined on 20 strains of clinical-isolated MDR-PA strains. Cinnamon bark oil showed the strongest activity against all MDR-PA isolates with MIC at 0.0562–0.225 % v/v and MBC at 0.1125–0.225 % v/v. Lemongrass oil ranked in the second with MIC at 0.45–1.8 % v/v and MBC varied from 0.9 up to higher than 1.8 % v/v, while clove oil showed MIC varied from 0.9 up to higher than 1.8 % v/v and MBC was more than 1.8 % v/v (Table 1).

**Table 1:** Susceptibility tests of cinnamon bark oil, lemongrass oil and clove oil against clinical-isolated MDR-PA strains

Isolated strain	Cinnamon bark oil		Clove oil		Lemongrass oil	
	MIC	MBC	MIC	MBC	MIC	MBC
	(% v/v)	(% v/v)	(% v/v)	(% v/v)	(% v/v)	(% v/v)
PA01	0.1125	0.225	1.8	>1.8	1.8	>1.8
PA04	0.1125	0.225	1.8	>1.8	1.8	>1.8
PA05	0.1125	0.225	>1.8	>1.8	0.9	0.9
PA07	0.225	1.8	1.8	1.8	1.8	1.8
PA12	0.1125	0.225	>1.8	>1.8	1.8	1.8
PA13	0.225	0.225	1.8	>1.8	1.8	1.8
PA16	0.1125	0.225	1.8	>1.8	0.9	1.8
PA17	0.225	0.225	>1.8	>1.8	1.8	>1.8
PA18	0.1125	0.225	1.8	>1.8	0.9	>1.8
PA19	0.1125	0.225	1.8	>1.8	1.8	>1.8
PA25	0.1125	0.1125	0.9	>1.8	0.45	1.8
PA26	0.1125	0.225	>1.8	>1.8	1.8	>1.8
PA31	0.1125	0.1125	1.8	>1.8	1.8	>1.8
PA34	0.1125	0.225	1.8	>1.8	0.9	>1.8
PA35	0.225	0.225	1.8	>1.8	1.8	>1.8
PA36	0.225	0.225	1.8	>1.8	1.8	>1.8
PA38	0.1125	0.225	1.8	>1.8	0.9	>1.8
PA41	0.0562	0.225	1.8	>1.8	1.8	>1.8
PA43	0.1125	0.225	1.8	>1.8	0.9	1.8
PA45	0.1125	0.1125	1.8	>1.8	1.8	>1.8

This study indicated that cinnamon bark oil demonstrated the most inhibitory effectiveness against clinical-isolated MDR-PA strains with MIC in range of 0.1125 to 0.225 % v/v which was consistent with the previous study that cinnamon oil was the strongest antimicrobial agent. The researchers demonstrated that clove and cinnamon oil possessed potent inhibitory effect on various bacterial pathogens. Moreover, cinnamon oil showed promising antimicrobial activity which could inhibit all tested bacterial strains both Gram-negative and Gram-positive bacteria with the lowest concentration among the total of 21 plant oils [22]. Warnke et al. [23] conducted a study including the activities of various essential oils using disc diffusion against MDR strains of *Staphylococcus* spp., *Streptrococcus* spp. and *Candida* spp. It revealed that thyme, lemon, lemongrass, cinnamon, tea tree, eucalyptus, grapefruit, clove bud, kunzea, sandalwood, peppermint, sage, and lavender oils affected on all tested microorganisms while olive and paraffin oil showed no activity. Essential oils from Thai medicinal plants including *Zingiber cassumuna*, *Cinnamomum bejolghota*, *Mentha arvensis* var. piperacens, *Cymbopogon citrates,* and *Ocimum basilicum* var. citratum demonstrated antibacterial activity against zoonotic enteropathogens including *Salmonella* spp., *E. coli* O157, *Campylobacter jejuni,* and *Clostridium perfringens* [24].

### Determination of the active compounds of cinnamon bark oil

**Fig. 1:**
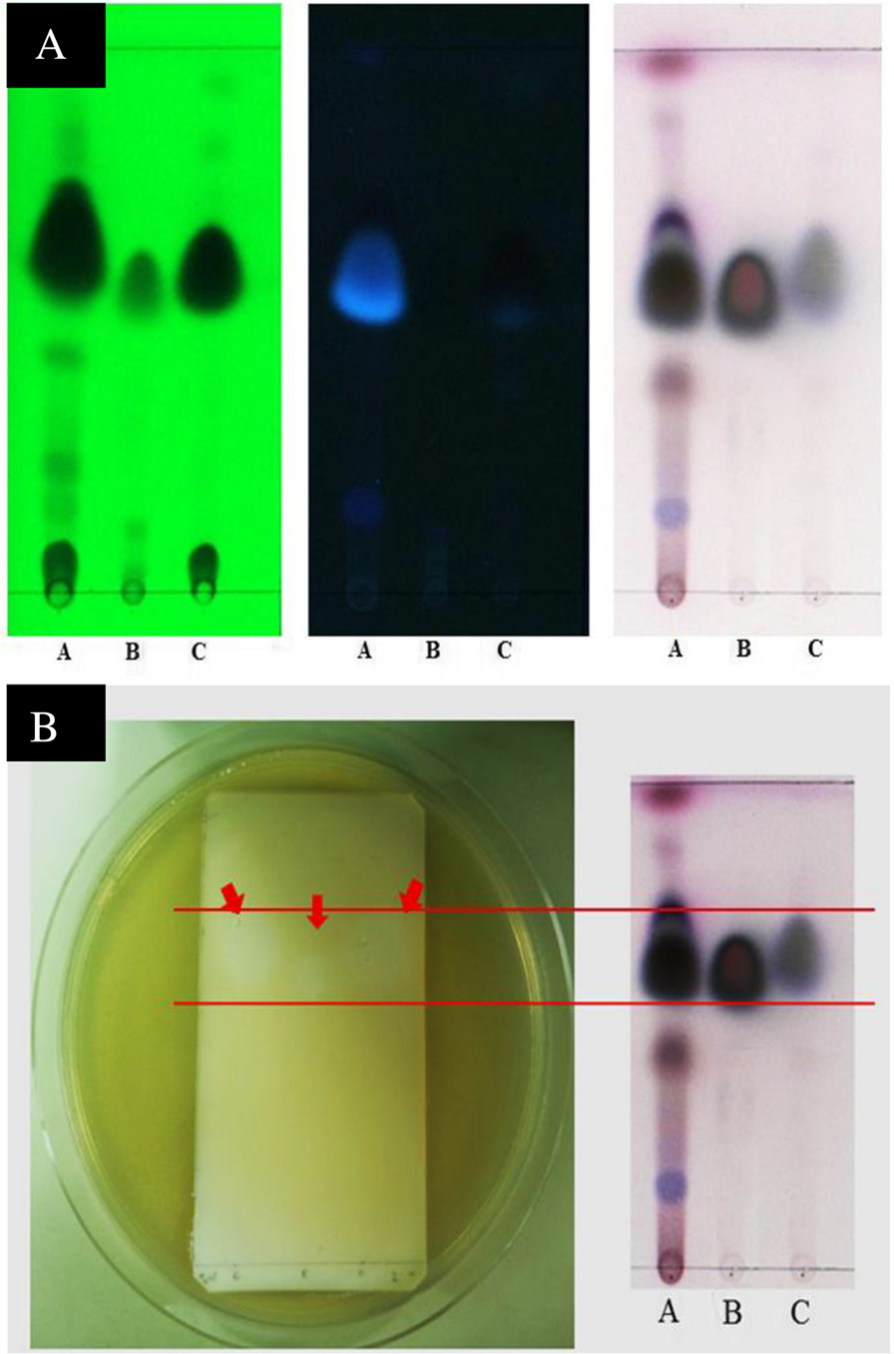
TLC chromatogram of cinnamon bark oil. TLC fingerprints of essential oils were developed by toluene and ethyl acetate with ratio of 93:7 v/v and visualized under UV light 254 nm (left), 366 nm (center) and visible light (right) (**a**). Bioautographic assay against *P. aeruginosa* was demonstrated (**b**). Lane A, cinnamon bark oil; lane B, eugenol; lane C, cinnamaldehyde. Arrows indicate inhibition zone by active compounds

The bioautographic assay was performed to determine the major active compounds of cinnamon bark oil. The results showed that cinnamon bark oil contained at least 8 compounds with the major active constituent having R_f_ of 0.6125 closely to the R_f_ of the cinnamaldehyde standard. Another component found in cinnamon bark oil was eugenol with R_f_ of 0.5625. The inhibition zones of cinnamon bark oil, eugenol and cinnamaldehyde against the growth of *P. aeruginosa* PAO1 and MDR-PA isolates were clearly shown. The clear zones locating separately on TLC suggested that cinnamon bark oil showed antibacterial activity similar to cinnamaldehyde while eugenol showed lower antibacterial activity than cinnamon bark oil (Fig. 1).

Cinnamaldehyde is considered to be an aromatic aldehyde substance found approximately 60–70 % in cinnamon bark oil [25]. The results of TLC and bioautographic assay in this study showed that the major compounds in cinnamon bark oil were mainly composed of cinnamaldehyde and eugenol. Cinnamaldehyde possessed better activity than eugenol against *P. aeruginosa*.

### Antibacterial activity of cinnamon bark oil and cinnamaldehyde under gaseous condition

Due to the nature of essential oils being odorous and volatile, antibacterial activities of the selected essential oils were also determined under vapor phase condition. Cinnamon bark oil and cinnamaldehyde demonstrated promising activity of gas phase against *P. aeruginosa* PAO1 and MDR-PA isolates with MID in a range of 0.5 to 1 mg/L air while eugenol, lemongrass and clove oil showed less inhibition activity with MID more than 1 mg/L air.

Under vapor condition, cinnamon bark oil and cinnamaldehyde also showed strong activity on MDR-PA. Some studies on vapor-phase antimicrobial activity of six essential oils on foodborne pathogens demonstrated that clove and cinnamon oil possessed more antibacterial activity than rosemary, basil, ginger, and dill oil against Gram-positive bacteria, Gram-negative bacteria, and three fungi in disc diffusion method [26]. Moreover, there was a study of antimicrobial effect of cinnamaldehyde on air-borne microbes. The study exhibited that cinnamaldehyde reduced germ count to 45 % after sprayed in the room [27].

### Time-killing effects of cinnamon bark oil and cinnamaldehyde

**Fig. 2:**
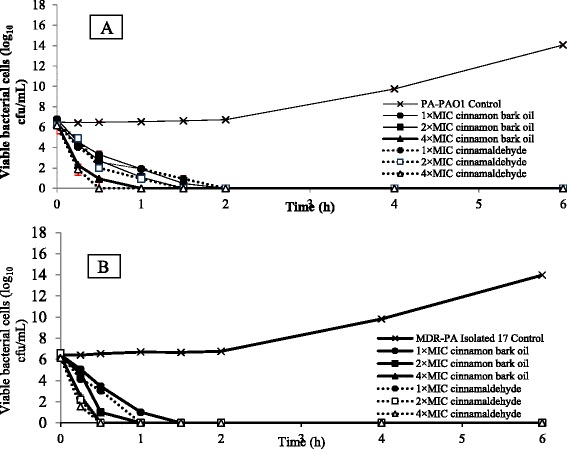
Time killing assay of cinnamon bark oil and cinnamaldehyde. *P. aeruginosa *PAO1 (**a**) and MDR-PA (**b**) were collected to observe viable cells after exposure to essential oils and incubated at 37 °C, 150 rpm. The results are expressed as mean values ± standard deviation of three independent experiments

*P. aeruginosa* PAO1 was challenged with cinnamon bark oil or cinnamaldehyde at various concentrations and bacterial viability was determined at different time points during incubation. The results showed that bacterial cells were killed within 1.3 and 2 h after treated with 1xMIC of cinnamaldehyde and cinnamon bark oil, respectively. Time-killing graphs demonstrated the reduction of viable cell approximately 6 log CFU/ml after *P. aeruginosa* PAO1 and MDR-PA contacted with the compound for 2 and 4 h, respectively (Fig. 2). Moreover, the higher concentrations of cinnamon oil and cinnamaldehyde (4xMIC) could efficiently eradicate MDR-PA within 1 h. These results suggested that cinnamon bark oil and cinnamaldehyde acted in dose-dependent manner on *P. aeruginosa*. Our study also reported that of cinnamon oil and its major compound at the studied concentrations showed significant effect on bacterial growth indicating dose-dependent inhibition activity in agreement with the group of Mayaud et al. [25].

### Synergistic effect of cinnamon bark oil/cinnamaldehyde and currently used antibiotics

*P. aeruginosa* PAO1 and 30 clinical isolates of MDR-PA were determined antimicrobial susceptibility with 7 antibiotics for MIC, MIC_50_ and MIC_90_ (Table 2). The results showed that COL had minimal MIC interval at 2–8 μg/ml. MIC_50_ and MIC_90_ of COL was 4 and 8 μg/ml, respectively. CAZ showed maximum MIC interval at 2 to more than 2,048 μg/ml. Interestingly, most MDR-PA isolates were resistant to MEM, CIP, and CAZ.

**Table 2:** Antimicrobial susceptibility of 30 clinically isolated MDR-PA strains against currently used antibiotics

Antibiotics	MIC range	MIC50	MIC90	% susceptibility		
	(μg/ml)	(μg/ml)	(μg/ml)	S	I	R
Colistin	2–8	4	8	23.3	63.4	13.3
Meropenem	1–1024	16	64	3.3	6.7	90
Imipenem	2–512	16	32	ND	16.7	83.3
Doripenem	0.5–256	4–8	16	3.3	43.3	53.4
Ciprofloxacin	0.5–128	64	128	10	ND	90
Piperacilin	2–1024	256	1024	36.7	ND	63.3
Ceftazidime	2– > 2048	>2048	>2048	6.7	3.3	90

*MIC range* MIC values from lowest to highest, *MIC50* MIC is position of percentile 50, *MIC90* MIC is position of percentile 90, *S* sensitive, COL (≤2 μg/ml), MEM (≤2 μg/ml), IMI (≤2 μg/ml), DOR (≤2 μg/ml), CIP (≤1 μg/ml), PIP (≤16 μg/ml), and CAZ (≤8 μg/ml), *I* intermediate, COL (4 μg/ml), MEM (4 μg/ml), IMI (4 μg/ml), DOR (4 μg/ml), CIP (2 μg/ml), PIP

Furthermore, synergistic interactions of ceftazidime, piperacillin, ciprofloxacin, meropenem, imipenem, doripenem, colistin and cinnamon bark oil or its major compound were further studied as a percentage of each couple combinations. Thirty MDR-PA isolates were tested in combination between antibiotics and cinnamon bark oil or its major active compound. Most of the combinations exhibited indifferent interaction of two active substances to bacterial isolates with FICI range of 0.5–4. Synergistic rates could be detected in some isolates which were at about 16.7 % in COL combined with cinnamon bark oil, 10 % in COL combined with cinnamaldehyde, and 3.3 % in MEM combined with cinnamon bark oil. It was noteworthy that the antagonistic activities were not be detected in any combinations (Table 3).

**Table 3:** Interaction between antibiotics and cinnamon bark oil or cinnamaldehyde against 30 clinically isolated MDR-PA strains

Combinations		% isolates of bacteria in each interaction activity		
		SYN	IND	ANT
Colistin	Cinnamon bark oil	16.7	83.3	ND
	Cinnamaldehyde	10	90	ND
Meropenem	Cinnamon bark oil	3.3	96.7	ND
	Cinnamaldehyde	ND	100	ND
Imipenem	Cinnamon bark oil	ND	100	ND
	Cinnamaldehyde	ND	100	ND
Doripenem	Cinnamon bark oil	ND	100	ND
	Cinnamaldehyde	ND	100	ND
Ciprofloxacin	Cinnamon bark oil	ND	100	ND
	Cinnamaldehyde	ND	100	ND
Piperacillin	Cinnamon bark oil	ND	100	ND
	Cinnamaldehyde	ND	100	ND
Ceftazidime	Cinnamon bark oil	ND	100	ND
	Cinnamaldehyde	ND	100	ND

*SYN* synergy, *IND* indifferent, *ANT* antagonist, *ND* not detected

Cinnamon bark oil and cinnamaldehyde possessed higher antibacterial activity of colistin and meropenem against MDR-PA strains. Cinnamon bark oil was suggested to act on bacterial membrane by dissipated potassium cation (K^+^) gradient leading to membrane damage and breakdown of permeability barrier resulting in cell death [28]. Domadia et al. described the mechanism of cinnamaldehyde which of bioactive compound was assembled into Z-ring at the site of cell separation by binding to FtsZ protein [29]. This data was an evidence to show a reduction of bacterial cell loading. Accumulation of various substances in cytoplasm and destruction of cytoplasmic membrane of bacteria could cause cellular substance leakage and cell death [30]. This might result in positive interaction in combination. Combination therapy is used for expanding the antimicrobial spectrum, reducing toxicity and decreasing the antimicrobial resistance during treatment. Moreover, the results in this work showed no regrowth of *P. aeruginosa* after exposed to cinnamon bark oil and trans-cinnamaldehyde over 24 h.

## Conclusions

Cinnamon bark oil is an essential oil obtained from the bark of *Cinnamomum zeylanicum* which belongs to Lauraceae family and usually grows in South and Southeast Asia. The current study reported that cinnamon bark oil and cinnamaldehyde possessed high bactericidal activity against MDR-PA isolates. Moreover, it showed promising tendency of combination with colistin, the currently used drug for treatment of gram-negative bacterial infection. Therefore, this could be a compound used for beneficial human health, considering as an alternative therapeutic agent for medical application and anti-bacterial supplement in health products, especially natural active compounds that might reduce the cost and could be safe. However, the further studies including the in vivo toxicity studies and clinical trials on cinnamon oil or its active compound to determine pharmacodynamics and pharmacokinetics are still necessary.

## Abbreviations

CAZ, ceftazidime; CIP, ciprofloxacin; COL, colistin sulfate; DOR, doripenem; FICI, fractional inhibitory concentration index; IMI, imipenem; MBCs, minimum bactericidal concentrations; MDR-PA, multidrug resistant *P. aeruginosa;* MEM, meropenem; MICs, minimum inhibitory concentrations; MID, minimum inhibitory dose; PIP, piperacillin; TLC, thin-layer chromatography.
